# Randomized, double-blind pilot study on botulinum toxin type A in the treatment of axillary hidradenitis suppurativa^؆^

**DOI:** 10.1016/j.abd.2026.501461

**Published:** 2026-09-04

**Authors:** Jessica Shen Tsy Wu Kim, Kevin Yun Kim, Laís de Abreu Mutti, Fernanda de Carvalho Tironi, Anamaria da Silva Facina, Samira Yarak, Sergio Henrique Hirata

**Affiliations:** Department of Dermatology, Universidade Federal de São Paulo, São Paulo, SP, Brazil

Dear Editor,

The pain associated with hidradenitis suppurativa (HS) can be intense and chronic, reported as the primary factor contributing to a decline in quality of life, followed by purulent discharge and appearance.^1^

Although HS is not considered a primary disease of the apocrine gland, it is believed that botulinum toxin type A—by temporarily blocking acetylcholine release—reduces apocrine secretion and limits follicular rupture. Consequently, there is reduced inflammation and a decrease in the number of nodules, abscesses, and fistulas, with remission maintained for approximately three to six months (the duration of the toxin's effect). The aim of this study was to evaluate the efficacy of botulinum toxin type A in treating axillary HS, assessing it as an accessible, effective therapeutic option with lower morbidity.

Following approval by the Research Ethics Committee, the study included patients over 18 years of age with Hurley stage II or III hidradenitis^2^ and lesions in both axillary regions for at least one year. All patients experienced episodes of inflammatory axillary lesions (abscesses and nodules) at least once a month, as confirmed by a clinical investigator.

Exclusion criteria included contraindications to botulinum toxin type A treatment; patients receiving systemic treatment within the previous three months; corticosteroid injection or surgical intervention within the four weeks prior to the study start; and treatment with botulinum toxin type A in the target area within the previous six months.

After randomization using the web-based program Random (www.random.org), one side was treated with an intradermal injection of toxin type A, while the other received an intradermal injection of sterile 0.9% saline solution. Both the patient and the physician administering the injections were blinded to the contents of the syringes.

Botulinum toxin type A (100 U)—diluted in 2 mL of 0.9% saline—was administered via 25 injection points of 4 U each, equivalent to 0.08 mL per point (with a spacing of 1–2.5 cm between points, corresponding to the toxin's halo of action). One mL syringes (UltraFine II, BD-Brazil, São Paulo, Brazil) and 30-gauge, 6 mm needles were used. Each dose was injected intradermally into the axilla, targeting both the lesions and the surrounding healthy skin. The same technique was applied to the contralateral axilla, injecting only 0.9% saline at the same volume and depth per point.

Assessments of the response to treatment and placebo were conducted by an independent researcher at weeks 2, 12, and 24 post-administration (visits 1, 2, and 3, respectively). No other interventions took place during the follow-up period. The parameters analyzed (participants achieving HiSCR at week 12,^3^ patient satisfaction,^4^ patient global assessment,^5^ and intensity of pain and secretion drainage) are presented in Table 1.^5^, ^6^, ^7^, ^8^

**Table 1:** Assessment scales and instruments used to measure response to treatment and placebo.

Analyzed parameters	Definition and scoring system
Participants who achieved “Hidradenitis Suppurativa Clinical Response” (HiSCR) at week 12^5^	Reduction of inflammatory lesions greater than or equal to 50% (sum of abscesses and inflammatory nodules); AND No increase in abscesses and draining fistulas compared to baseline.

Patient satisfaction level^6^, ^7^	Those with a score of +1 are considered responders:
	-1 = dissatisfied, with worsening compared to the start of treatment, not recommending the treatment
	0 = indifferent to treatment, with the same situation at the beginning of treatment
	+1 = satisfied, better than at the start of treatment; would recommend the treatment and undergo it again if necessary.

Patient Global Assessment^8^	Those with a score ≥+2 are considered responders:
	-3 = much worse than before the treatment
	-2 = moderately worse (approximately 50% more disease activity)
	-1 = a little worse
	0 = the same as before the treatment
	+1 = a little better
	+2 = moderately better (approximately 50% reduction in disease activity)
	+3 = much better (no disease activity or almost no disease activity)

Report on pain intensity and secretion drainage	Visual Analog Scale (VAS) with a 0–10 cm vertical line, combined with the Wong-Baker Scale, where 0 corresponds to the absence of pain or low intensity, and 10 corresponds to severe pain or maximum intensity.
	Responders were defined as those who achieved at least a 30% reduction and a 1 cm absolute reduction in pain score compared to baseline.

Statistical analysis was performed using McNemar's test to evaluate paired nominal data. An alpha significance level of 5% was adopted, and R software version 4.4.0 (2024-04-24 UCRT) was used for the analyses.

The initial sample consisted of 16 patients, predominantly female (75%), with a mean age of 32 years. Regarding self-reported race/ethnicity, 50% were mixed-race (brown), 31.25% were white, and 18.75% were black. The cause of HS is unknown, but it is believed to be multifactorial. Studies identify heredity and smoking as risk factors.^6^ In the present study, 25% of the patients had a positive family history (35%–40% in the literature),^7^ and current or past smoking was present in 37.5% of the sample (up to 90% in the literature).^8^ In this protocol, only one patient was not overweight or obese. Half of the individuals (50%) had severe acne, 25% had pilonidal cysts, 12.5% ​​ had abscessing folliculitis, and 12.5% ​​had systemic arterial hypertension. There were no cases of inflammatory bowel disease, spondyloarthropathy, or pyoderma gangrenosum in the analyzed sample.

During the protocol, two patients (12.5%) failed to attend any of the three visits and were excluded from statistical analyses. All patients who attended each of their respective visits were included.

As shown in Table 2, the assessment of clinical outcomes at visits 1, 2, and 3 showed no statistically significant differences between the intervention and placebo groups for any of the analyzed variables (all p-values ​​> 0.05). At visit 1, a similar distribution of HiSCR response was observed between the groups, as well as comparable results regarding patient satisfaction, global assessment, pain, and drainage. At visit 2, the findings remained equivalent, notably with a non-significant trend toward greater pain reduction in the placebo group (p = 0.063). By the third visit, the absence of differences between the groups persisted, with numerical trends even favoring the placebo group regarding subjective outcomes, such as satisfaction and global assessment.

**Table 2:** Assessment of response to treatment and placebo at each of the visits conducted 2, 12, and 24 weeks after application (visits 1, 2, and 3, respectively).

Visit 1: n = 13 patients (26 axillae)				
Variable	Categories	Intervention	Placebo	p-value
HiSCR	Yes	3 (16.6%)	3 (16.6%)	1
	No	6 (33.3%)	6 (33.3%)	
	Does not apply^a^: 8 axillae			
Patient satisfaction:	Yes	9 (34.6%)	6 (23%)	0.375
	No	4 (15.3%)	7 (26.9%)	
Global assessment:	Yes	5 (19.2%)	2 (7.6%)	0.25
	No	8 (30.7%)	11 (42.3%)	
VAS pain:	Yes	9 (36%)	5 (20%)	0.25
	No	4 (16%)	7 (28%)	
	Does not apply^b^: 1 axilla			
VAS drainage	Yes	7 (31%)	5 (22.7%)	1
	No	5 (22.7%)	5 (22.7%)	
	Does not apply^b^: 4 axillae			

Visit 2: n = 14 patients (28 axillae)				
Variable	Categories	Intervention	Placebo	p-value
HiSCR	Yes	2 (10.5%)	3 (15.7%)	1
	No	8 (42.1%)	6 (31.5%)	
	Does not apply^a^: 9 axillae			
Patient satisfaction	Yes	10 (35.7%)	10 (35.7%)	1
	No	4 (14.2%)	4 (14.2%)	
Global assessment	Yes	5 (17.8%)	6 (21.4%)	1
	No	9 (32.1%)	8 (28.5%)	
VAS pain	Yes	7 (25.9%)	9 (33.3%)	0.063
	No	7 (25.9%)	4 (14.8%)	
	Does not apply^b^: 1 axilla			
VAS drainage	Yes	6 (25%)	6 (25%)	1
	No	7 (29.1%)	5 (20.8%)	
	Does not apply^b^: 4 axillae			

Visit 3: n = 10 patients (20 axillae)				
Variable	Categories	Intervention	Placebo	p-value
HiSCR	Yes	0	1 (8.3%)	1
	No	6 (50%)	5 (41.7%)	
	Does not apply^a^: 8 axillae			
Patient satisfaction	Yes	5 (25%)	8 (40%)	0.375
	No	5 (25%)	2 (10%)	
Global assessment	Yes	2 (10%)	5 (25%)	0.375
	No	8 (40%)	5 (25%)	
VAS pain	Yes	4 (21%)	5 (26.3%)	0.5
	No	6 (31.5%)	4 (21%)	
	Does not apply^b^: 1 axilla			
VAS drainage	Yes	6 (37.5%)	4 (25%)	1
	No	3 (18.75%)	3 (18.75%)	
	Does not apply^b^: 4 axillae			

VAS, Visual Analog Scale.

a Axillae with fewer than 3 inflammatory lesions at baseline.

b Axillae with a score of less than 10 mm on the VAS for pain or drainage at baseline.

This study demonstrated no statistically significant difference between Botulinum Toxin Type A (BTX-A) and the placebo (0.9% saline) for any of the evaluated outcomes—including secretion—at any of the three visits. This lack of superiority for the active intervention, while initially surprising, must be interpreted within the context of an exploratory study featuring a small sample size (n = 14 after dropouts), an interpatient design, and a disease characterized by a cyclical and multifactorial nature.

Botulinum toxin, already used to treat hyperhidrosis, represents an innovative approach with a plausible biological rationale: it temporarily blocks acetylcholine release, thereby reducing moisture and altering axillary microbiota. This process may decrease the formation of inflammatory lesions by reducing apocrine secretion and limiting follicular rupture.^9^ Consequently, there is potential for reduced inflammation and a decrease in the number of nodules, abscesses, and fistulas, as well as the maintenance of remission. Although the effect of BTX-A is limited in duration—requiring repeated applications—the intervention has an excellent safety profile. If its efficacy is confirmed, it could significantly improve the quality of life for patients refractory to conventional therapies, thereby avoiding invasive surgical procedures and the high costs associated with biologic agents.

Case reports and a recent systematic review^9^ compiled data on 31 patients with hidradenitis suppurativa (ranging from Hurley stages I, II, and/or III, or with the Hurley stage unreported) treated with botulinum toxin A or B; 96.8% of these patients showed clinical improvement or an improvement in quality of life. These data are promising but limited by methodological heterogeneity, small sample sizes, and the absence of placebo controls in most cases. The only previous randomized, double-blind trial using toxin type B^10^ also failed to demonstrate significant superiority, although it did report improvements in DLQI scores and a trend toward lesion reduction in subgroups, which suggests that differences between toxin types A and B might influence outcomes in HS.

In conclusion, although intradermal BTX-A did not prove superior to placebo in this pilot study, the findings do not rule out its therapeutic potential. The intervention remains a promising alternative—offering easy outpatient administration and a favorable safety profile—particularly for patients who are refractory to conventional treatments or ineligible for biologics. Further randomized, controlled trials with greater statistical power and optimized study designs are essential to clarify the role of this treatment within the therapeutic arsenal for HS.

## ORCID ID

Jessica Shen Tsy Wu Kim: 0000-0003-4133-6689

Kevin Yun Kim: 0000-0003-0727-6539

Laís de Abreu Mutti: 0000-0002-9410-1958

Fernanda de Carvalho Tironi: 0000-0002-1985-6795

Anamaria da Silva Facina: 0000-0002-2888-7227

Sergio Henrique Hirata: 0000-0003-4026-9664

## Research data availability

The entire dataset supporting the results of this study was published in this article.

## Financial support

This study was conducted with financial support from FUNADERSP (*Fundo de Apoio à Dermatologia do Estado de São Paulo*), project number 75-2018.

## Authors' contributions

Jessica Shen Tsy Wu Kim: Conceptualization; data analysis and curation; literature review; drafting and editing of the manuscript; critical review of relevant intellectual content; approval of the final version of the manuscript.

Kevin Yun Kim: Conceptualization; data analysis and curation; literature review; drafting and editing of the manuscript; critical review of relevant intellectual content; approval of the final version of the manuscript.

Laís de Abreu Mutti: Conceptualization; data analysis and curation; literature review; drafting and editing of the manuscript.

Fernanda de Carvalho Tironi: Conceptualization; data analysis and curation; critical review of the literature.

Anamaria da Silva Facina: Critical review of relevant intellectual content; drafting and editing of the manuscript; effective supervision and research orientation.

Samira Yarak: Critical review of relevant intellectual content; drafting and editing of the manuscript; effective supervision and research orientation; approval of the final version of the manuscript.

Sergio Henrique Hirata: Conceptualization; critical review of relevant intellectual content; effective supervision and research orientation; approval of the final version of the manuscript.

## Conflicts of interest

None declared.
